# Synthesis and Biological Screening of 5-{[(4,6-Disubstituted pyrimidine-2-yl)thio]methyl}-N-phenyl-1,3,4-thiadiazol-2-amines

**DOI:** 10.4103/0250-474X.45416

**Published:** 2008

**Authors:** M. A. Azam, B. R. P. Kumar, S. Shalini, B. Suresh, T. K. Reddy, C. D. Reddy

**Affiliations:** Department of Pharmaceutical Chemistry, J. S. S. College of Pharmacy, Ootacamund-643 001, India; 1 Sugen Life Sciences, A Division of Cancer Biology, Tirupati-517 505, India

**Keywords:** Thiadiazoles, pyrimidines, chalcones, thiourea and anticancer activity

## Abstract

A number of substituted-α,β-unsaturated carbonyl compounds (1a-i) were prepared by Claisen-Schmidt condensation of substituted acetophenone with selected araldehydes, which on cycloaddition with thiourea furnished 4,6-disubstituted pyrimidine-2-thiols (2a-i). Reaction of (2a-i) with ethyl chloroacetate followed by condensation with hydrazine hydrate yielded 2-[(4,6-disubstituted pyrimidine-2-yl) thio] acetohydrazides (4a-c). Condensation of compounds (4a-c) with phenyl isothiocyanate gave 2-{[(4,6-disubstituted pyrimidine-2-yl) thio] acetyl}-N-phenylhydrazinecarbothioamides (5a-c) which on treatment with concentrated sulphuric acid afforded titled compounds 5-{(4,6-disubstituted pyrimidine-2-yl) thio] methyl}-N-phenyl-1,3,4-thiadiazole-2-amines (6a-c). These compounds have been characterized on the basis of elemental analysis, IR, ^1^H NMR and MS. Compounds have been evaluated for their anticancer and antioxidant activities. Compounds 2b, 2c and 6b exhibited significant antitumor activity against human breast cancer MCF 7 cell line. However, moderate antioxidant activity was observed with compounds 2c, 2d, 2g and 6b.

In recent years pyrimidine derivatives have received significant attention owing to their diverse range of biological properties particularly being antifungal^1^, antitubercular^2^, antibacterial^3^,^4^, antiviral^5^–^8^, anticancer^9^ and antioxidant^10^. 2,5-Disubstituted-1,3,4-thiadiazoles represent one of the most active classes of compounds possessing wide spectrum of biological activities. 2,5-Disubstituted-1,3,4-thiadiazole derivatives exhibit *in vitro* antimycobacterial^11^, antibacterial^12^, anticancer^13^,^14^ and antioxidant^15^ properties. Considering the above facts, the goal of the present study was to combine disubstituted pyrimidines with 1,3,4-thiadiazole residues in order to develop hybrid molecules with potential of enhanced activity and to test their antioxidant and antitumor activities.

Melting points were taken in open capillary tubes and are uncorrected. The IR spectra (KBr, cm^−1^) were recorded on a Shimadzu FTIR 800 series spectrophotometer and ^1^H NMR spectra (CDCl_3_) on Varian EM 390 MHz spectrometer using TMS as internal standard. Mass spectra were recorded on Shimadzu 2010A LC-MS system. The reactions were monitored by thin layer chromatography using silica gel plates and detected by UV chamber and iodine as visualizing agent. The purity of the compounds was checked on silica gel precoated plates. All the solvents used were purified according to the standard methods^16^. Phenyl isothiocyanate was prepared according to the standard method^17^.

For the preparation of 4, 6-disubstituted pyrimidine-2-thiols (2a-i) a mixture of appropriate chalcones (1a-i, Scheme 1) (0.01 mol) and thiourea (0.01 mol) in ethanol (50 ml) and sodium hydroxide (0.01 mol) dissolved in minimum quantity of water was refluxed on a water bath for 12 h and poured into 250 ml of cold water. The solid that separated in each case was filtered, washed with water and recrystallized from ethyl acetate (Table 1); 2a: IR (KBr, cm^−1^): 3095 (aromatic C-H str.), 2830 (S-H str.), 1640 (C=N), 1590, 1610 (aromatic C=C str.), 1520 (C-N str.); MS: m/z 264 (M^+^); Anal. Calcd. for C_16_H_12_N_2_S: C, 72.72; H, 4.54; N, 10.60. Found: C, 72.75; H, 4.58; N, 10.56%; 2b: IR (KBr, cm^−1^): 3120 (aromatic C-H str.), 2840 (S-H str.), 1651 (C=N), 1582, 1606 (aromatic C=C str.), 1516 (C-N str.), 1265 (C-O-C); ^1^H NMR (CDCl_3_): δ 9.72 (s, 1H, SH), 6.81-8.32 (m, 11H, aromatic and heterocyclic), 3.75 (s, 3H, OCH_3_); MS: m/z 294 (M^+^); Anal. Calcd. for C_17_H_14_N_2_OS: C, 69.38; H, 4.76; N, 9.52. Found: C, 69.37; H, 4.81; N, 9.58%; 2c: IR (KBr, cm^−1^): 3330 (OH), 3088 (aromatic C-H str.), 2842 (S-H str.), 1649 (C=N), 1608 (aromatic C=C str.), 1518 (C-N str.); MS: m/z 280 (M^+^); Anal. Calcd. for C_16_H_12_N_2_OS: C, 68.57; H, 4.28; N, 10.00. Found: C, 68.61; H, 4.31; N, 9.97%; 2d: IR (KBr, cm^−1^): 3360 (OH), 3082 (aromatic C-H str.), 2835 (S-H str.), 1635 (C=N), 1580, 1608 (aromatic C=C str.), 1524 (C-N str.), 1280 (C-O-C); MS: m/z 310 (M^+^); Anal. Calcd. for C_17_H_14_N_2_SO_2_: C, 65.80; H, 4.51; N, 9.03. Found: C, 65.68; H, 4.54; N, 9.10%; 2e: IR (KBr, cm^−1^): 3082 (aromatic C-H str.), 2820 (S-H str.), 1635 (C=N), 1580, 1608 (aromatic C=C str.), 1524 (C-N str.), 1345 (NO_2_), 1275 (C-O-C); MS: m/z 339 (M^+^); Anal. Calcd. for C_17_H_13_N_3_O_3_S: C, 60.17; H, 3.83; N, 12.38. Found: C, 60.12; H, 3.85; N, 12.33%; 2f: IR (KBr, cm^−1^): 3328 (OH), 3086 (aromatic C-H str.), 2840 (S-H str.), 1644 (C=N), 1580, 1605 (aromatic C=C str.), 1524 (C-N str.); MS: m/z 280 (M^+^); Anal. Calcd. for C_16_H_12_N_2_ OS: C, 68.57; H, 4.28; N, 10.00. Found: C, 68.63; H, 4.34; N, 9.93%; 2g: IR (KBr, cm^−1^): 3330 (OH), 3060 (aromatic C-H str.), 3010 (C=C, alkene), 2825 (S-H str.), 1615 (C=N), 1598 (aromatic C=C str.), 1524 (C-N str.); MS: m/z 306 (M^+^); Anal. Calcd. for C_18_H_14_N_2_OS : C, 70.58; H, 4.57; N, 9.15. Found: C, 70.60; H, 4.53; N, 9.19%; 2h: IR (KBr, cm^−1^): 3310 (OH), 3072 (aromatic C-H str.), 2833 (S-H str.), 1618 (C=N), 1585 (aromatic C=C str.), 1520 (C-N str.), 1105 (C-O-C); MS: m/z 270 (M^+^); Anal. Calcd. for C_14_H_10_N_2_O_2_S: C, 62.22; H, 3.70; N, 10.37. Found: C, 62.28; H, 3.67; N, 10.29%; 2i: IR (KBr, cm^−1^): 3075 (aromatic C-H str.), 2830 (S-H str.), 1610 (C=N), 1605 (aromatic C=C str.), 1522 (C-N str.), 1352 (NO_2_); MS: m/z 334 (M^+^); Anal. Calcd. for C_18_H_12_N_3_O_2_S : C, 64.67; H, 3.59; N, 12.57. Found: C, 64.60; H, 3.62; N, 12.61%.

**Scheme 1:**
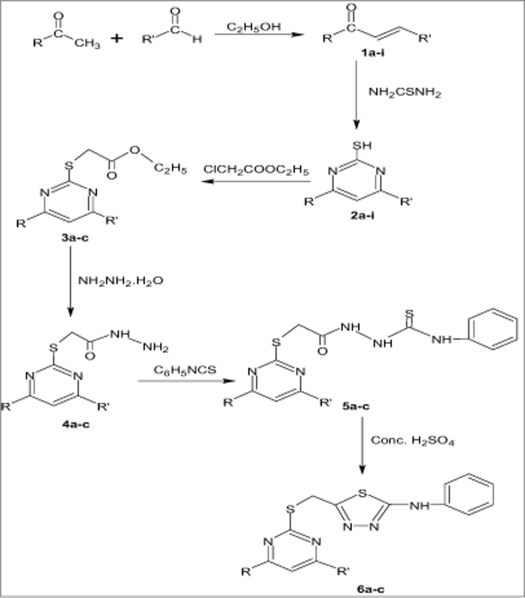
Synthesis of {[(4,6-disubstitutedpyrimidine-2-yl)thio] methyl}-N-phenyl-1,3,4-thiadiazol-2-amine R= -C_6_H_5_, 2-OH.C_6_H_4_ and 4-NO_2_.C_6_H_4_, R'= -C_6_H_5_, 4-OCH_3_.C_6_H_4_, 2-OH. C_6_H_4_, -CH=CH.C_6_H_5_ and 3-furyl

**TABLE 1:** CHARACTERIZATION DATA OF SYNTHESIZED COMPOUNDS^d^

Compd.	R	R'	Mol. Formula	M.P. °C	%Yield^a^
1a	C_6_H_5_	C_6_H_5_	C_15_H_12_O	57	80
1b	C_6_H_5_	4-OCH_3_.C_6_H_4_	C_16_H_14_O_2_	72	85
1c	C_6_H_5_	2-OH.C_6_H_4_	C_15_H_12_O_2_	66	81
1d	2-OH.C_6_H_4_	4-OCH_3_.C_6_H_4_	C_6_H_14_O_3_	75	78
1e	4-NO_2_.C_6_H_4_	4-OCH_3_.C_6_H_4_	C_16_H_13_O_4_N	77	65
1f	2-OH.C_6_H_4_	C_6_H_5_	C_15_H_12_O_2_	60	85
1g	2-OH.C_6_H_4_	C_6_H_5_CH=CH-	C_17_H_14_O_2_	69	55
1h	2-OH.C_6_H_4_	3-furyl	C_13_H_10_O_3_	82	67
1i	4-NO_2_.C_6_H_4_	C_6_H_5_.CH=CH-	C_17_H_13_O_3_N	78	60
2a	C_6_H_5_	C_6_H_5_	C_16_H_12_N_2_S	165	81
2b	C_6_H_5_	4-OCH_3_.C_6_H_4_	C_17_H_14_N_2_OS	80	75
2c	C_6_H_5_	2-OH.C_6_H_5_	C_16_H_12_N_2_OS	130	56
2d	2-OH.C_6_H_4_	4-OCH_3_.C_6_H_4_	C_17_H_14_N_2_O_2_S	120	84
2e	4-NO_2_.C_6_H_4_	4-OCH_3_.C_6_H_4_	C_17_H_13_N_3_O_3_S	140	96
2f	4-OH.C_6_H_4_	C_6_H_5_	C_16_H_12_N_2_OS	175	89
2g	2-OH.C_6_H_4_	C_6_H_5_.CH=CH-	C_18_H_14_N_2_OS	265	77
2h	2-OH.C_6_H_4_	3-Furyl	C_14_H_10_N_2_O_2_S	101	82
2i	4-NO_2_.C_6_H_4_	C_6_H_5_.CH=CH-	C_18_H_12_N_3_O_2_S	270	63
3a	C_6_H_5_	C_6_H_5_	C_20_H_18_N_2_O_2_S	118	86
3b	C_6_H_5_	4-OCH_3_.C_6_H_4_	C_21_H_20_N_2_O_3_S	198	65
3c	C_6_H_5_	2-OH.C_6_H_4_	C_20_H_18_N_2_O_3_S	212	57
4a	C_6_H_5_	C_6_H_5_	C_18_H_16_N_4_OS	202	65
4b	C_6_H_5_	4-OCH_3_.C_6_H_4_	C_19_H_18_N_4_O_2_S	199	55
4c	C_6_H_5_	2-OH.C_6_H_4_	C_18_H_16_N_4_O_2_S	215	61
5a	C_6_H_5_	C_6_H_5_	C_25_H_21_N_5_OS_2_	186	62
5b	C_6_H_5_	4-OCH_3_.C_6_H_4_	C_26_H_23_N_5_O_2_S_2_	189	53
5c	C_6_H_5_	2-OH.C_6_H_4_	C_25_H_21_N_5_O_2_S_2_	175	64
6a	C_6_H_5_	C_6_H_5_	C_25_H_19_N_5_S_2_	198	56
6b	C_6_H_5_	4-OCH_3_.C_6_H_4_	C_26_H_21_N_5_OS_2_	210	51
6c	C_6_H_5_	2-OH.C_6_H_4_	C_25_H_19_N_5_OS_2_	235	58

a Isolated yield, compounds 1a-i were synthesized by the known procedure^21^

d all compounds showed satisfactory elemental analysis

Preparation of ethyl [(4,6-disubstituted pyrimidine-2-yl) thio] acetates (3a-c) was achieved by mixing equimolar quantities of 4,6-disubstituted pyrimidine-2-thiols (2a-c) (0.017 mol), ethyl chloroacetate (2.017 g, 0.017 mol) and anhydrous potassium carbonate (1.10 g, 0.01 mol) in dry acetone (15 ml) and refluxing on a water bath for about 13 h. The mixture was then diluted with benzene and washed with water. The organic layer was dried (Na_2_SO_4_) and the solvent was removed under reduced pressure. The resulting solid in each case was recrystallized from benzene:petroleum ether (1:1) (Table 1); 3a: IR (KBr, cm^−1^): 3065 (aromatic C-H str.), 2910, 2886 (aliphatic C-H str.), 1745 (>C=O of ester), 712 (C-S-C); MS m/z: 350 (M^+^); Anal. Calcd. for C_20_H_18_N_2_O_2_S: C, 68.57; H, 5.14; N, 8.00. Found: C, 68.61; H, 5.18; N, 7.95%; 3b: IR (KBr, cm^−1^): 3061 (aromatic C-H str.), 2912, 2875 (aliphatic C-H str.), 1736 (>C=O of ester), 1240 (C-O-C), 710 (C-S-C); ^1^H NMR (CDCl_3_): δ 6.82-8.10 (m, 10H, aromatic and heterocyclic), 4.32 (q, 2H, COOCH_2_CH_3_), 4.12 (s, 2H, S-CH_2_-CO), 3.72 (s, 3H, OCH_3_), 1.05 (t, 3H, -COOCH_2_CH_3_); MS m/z: 380 (M^+^); Anal. Calcd. for C_21_H_20_N_2_O_3_S: C, 66.31; H, 5.26; N, 7.36. Found: C, 66.25; H, 5.32; N, 7.29%; 3c: IR (KBr, cm^−1^): 3320 (OH), 3070 (aromatic C-H str.), 2930, 2885 (aliphatic C-H str.), 1742 (>C=O of ester), 715 (C-S-C); MS m/z: 366 (M^+^); Anal. Calcd. for C_20_H_18_N_2_O_3_S: C, 65.57; H, 4.91; N, 7.65. Found: C, 65.63; H, 5.14; N, 7.58%.

For preparation of 2-[(4,6-disubstituted pyrimidine-2-yl) thio] acetohydrazides (4a-c), a solution of the appropriate esters (3a-c) (0.01 mol), hydrazine hydrate (3.5 ml) and ethanol (25 ml) was refluxed on a water bath for about 10 h. The solvent was then removed under reduced pressure and the residue obtained in each case was recrystallized from methanol (Table 1); 4a: IR (KBr, cm^−1^): 3420, 3375 (NH-NH_2_) 1660 (>C=O, amido), 1622 (C=N str.), 1605 (aromatic C=C), 715 (C-S-C); MS m/z: 336 (M^+^); Anal. Calcd. for C_18_H_16_N_4_OS: C, 64.28; H, 4.76; N, 16.66. Found: C, 64.33; H, 4.69; N, 16.71%; 4b: IR (KBr, cm^−1^): 3438, 3380, 3310 (NH-NH_2_) 1653 (>C=O, amido), 1642 (C=N str.), 1598 (aromatic C=C), 1250 (C-O-C), 712 (C-S-C); ^1^H NMR (CDCl_3_): δ 9.51 (s, 1H, CONH), 6.82-7.91 (m, 10H, aromatic and heterocyclic), 6.49 (bs, 2H, NH_2_), 4.20 (s, 2H, S-CH_2_-CO), 3.71 (s, 3H, OCH_3_); MS m/z: 366 (M^+^); Anal. Calcd. for C_19_H_18_N_4_O_2_S: C, 62.29; H, 4.92; N, 15.30. Found: C, 62.31; H, 4.87; N, 15.29%; 4c: IR (KBr, cm^−1^): 3380 (OH), 3345, 3320 (NH-NH_2_), 1668 (>C=O, amido), 1622 (C=N str.), 1598 (aromatic C=C), 711 (C-S-C); MS m/z: 352 (M^+^); Anal. Calcd. for C_18_ H_16_N_4_O_2_S: C, 61.36; H, 4.54; N, 15.90. Found: C, 61.41; H, 4.59; N, 15.83%.

For preparation of 2-[(4, 6-disubstituted pyrimidine-2-yl) thio] acetyl-N-phenylhydrazine carbothiamide (5a-c), a mixture of the acid hydrazides (4a-c) (0.01 mol) and phenylisothiocyanate (0.0015 mol) in ethanol (10 ml) was refluxed on a water bath for about 8 h. The solution was allowed to reach ambient temperature and the resulting solid in each case was collected and recrystallized from methanol (Table 1); 5a: IR (KBr, cm^−1^): 3225, 3215, 3180 (N-H), 3035, (aromatic C-H str.), 1670 (>C=O), 1625 (C=N), 1605 (aromatic C=C str.), 1450 (>C=S), 718 (C-S-C); MS m/z: 471 (M^+^); Anal. Calcd. for C_25_H_21_N_5_OS_2_: C, 63.69; H, 4.45; N, 14.86. Found: C, 63.72; H, 4.37; N, 14.79%; 5b: IR (KBr, cm^−1^): 3120-3218 (N-H), 3024 (aromatic C-H str.), 1681 (>C=O), 1667 (C=N), 1605 (aromatic C=C str.), 1453 (>C=S), 1255 (C-O-C), 710 (C-S-C); ^1^H NMR (CDCl_3_): δ 8.20-10.12 (m, 3H, NH.NH.CS.NH), 7.21-7.92 (m, 15H, aromatic and heterocyclic), 4.21(s, 2H, S-CH_2_-CO), 3.48 (s, 3H, OCH_3_); MS m/z: 501 (M^+^); Anal. Calcd. for C_26_H_23_N_5_O_2_S_2_: C, 62.27; H, 4.59; N, 13.97. Found: C, 61.98; H, 4.48; N, 13.89%; 5c: IR (KBr, cm^−1^): 3340 (OH), 3218, 3205, 3185 (N-H), 3045, (aromatic C-H str.), 1695 (>C=O), 1625 (C=N), 1598 (aromatic C=C str.), 1448 (>C=S), 714 (C-S-C); MS m/z: 487 (M^+^); Anal. Calcd. for C_25_H_21_N_5_O_2_S_2_: C, 61.60; H, 4.31; N, 14.37. Found: C, 61.67; H, 4.39; N, 14.31%.

For preparation of 5-{[(4,6-disubstituted pyrimidine-2-yl) thio] methyl}-N-phenyl-1,3,4-thiadiazol-2-amine (6a-c), a mixture of the appropriate thiosemicarbazides (5a-c) (0.001 mol) in cold concentrated sulphuric acid (3 ml) was stirred for 10 min the resulting solution was then allowed to reach ambient temperature and poured cautiously into ice cold water. The reaction mixture was made alkaline to pH 8 with aqueous ammonia and the precipitated product in each case was collected washed with cold water and recrystallized from ethanol (Table 1); 6a: 3395 (N-H), 3095 (aromatic C-H str.), 2955 (C-H str.), 1620 (C=N, str.), 1605 (aromatic C=C str.), 740 (C-S-C, thiadiazole), 710 (C-S-CH_2_); MS m/z: 453 (M^+^); Anal. Calcd. for C_25_H_19_N_5_S_2_: C, 66.22; H, 4.19; N, 15.45. Found: C, 66.29; H, 4.14; N, 15.39%; 6b: 3440 (N-H), 3211 (aromatic C-H str.), 2939 (C-H str.), 1665 (C=N, str.), 1599 (aromatic C=C str.), 1248 (C-O-C), 746 (C-S-C, thiadiazole), 708 (C-S-CH_2_); ^1^H NMR (CDCl_3_): δ 9.3 (s, 1H, NH), 7.21-8.32 (m, 15H, aromatic and heterocyclic), 3.38 (s, 3H, OCH_3_), 3.61 (s, 2H, -CH_2_-); MS m/z: 483 (M^+^); Anal. Calcd. for C_26_H_21_N_5_OS_2_: C, 64.59; H, 4.34; N, 14.49. Found: C, 64.61; H, 4.42; N, 14.51%; 6c: 3380 (N-H), 3345 (OH), 3070 (aromatic C-H str.), 2975 (C-H str.), 1612 (C=N, str.), 1610 (aromatic C=C str.), 726 (C-S-C, thiadiazole), 712 (C-S-C); MS m/z: 469 (M^+^); Anal. Calcd. for C_25_H_19_N_5_OS_2_: C, 63.96; H, 4.05; N, 14.92. Found: C, 64.11; H, 4.09; N, 14.89%.

Determination of *In vitro* antioxidant activity was done by DPPH (1,1-diphenyl-2-picrylhydrazyl)^18^ and nitric oxide^19^ free radical scavenging methods. The methods were used to screen compounds (2a-i) and (6a-c) for the antioxidant activity. Ascorbic acid and rutin were used as reference standards at a concentration level of 100 μg/ml. Results are presented in Table 2.

**TABLE 2:** *IN VITRO* ANTICANCER AND ANTIOXIDANT ACTIVITIES OF COMPOUNDS (2A-I) AND (6A-C)

Compd.	Antioxidant activity		Average percent growth values** at 20 μM in MCF-7 cell line
	
	DPPH method IC_50_ (μg/ml)*	Nitric oxide method IC_50_ (μg/ml)*		
2a	>500	105	67.77
2b	>500	160	13.33
2c	56	95	0
2d	50.5	90	79.98
2e	84	>500	34.44
2f	>500	125	94.44
2g	60	200	77.50
2h	84	>500	43.55
2i	>500	>500	97.4
6a	82	143	68.54
6b	92	87	12.55
6c	78	>500	58.50
	18	69	--
	(ascorbic acid)	(rutin)	

* Average of three determinations, both test compounds and standard were tested at 100 μg/ml, IC_50_ concentration of the test compound causing 50% decrease of activity against control.

** Mean of two determinations, ^a^zero indicates that no cells have died.

Antitumor activity of the compounds was evaluated by tryphan blue dye exclusion technique^20^ against human breast cancer MCF-7 cell line at 20 μM concentration. Primary screening of the compounds was done to indicate whether a substance possessed enough activity at this concentration to inhibit cell growth by 50%. Results are presented in Table 2.

Chalcones (1a-i) required as starting material were prepared^21^ by stirring equimolar solution of various substituted acetophenones and araldehydes in the presence of sodium hydroxide in ethanol at room temperature (Scheme 1). Solution in ethanol of chalcones (1a-i) and thiourea in the presence of sodium hydroxide was refluxed on a water bath to yield 4,6-disubstituted pyrimidine-2-thiols (2a-i). When thiols (2a-c) and ethyl chloroacetate was refluxed in the presence of anhydrous sodium carbonate resulted in the formation of ethyl [(4,6-disubstituted pyrimidine-2-yl) thio] acetates (3a-c). Compounds (3a-c), hydrazine hydrate in ethanol as a reaction media afforded 2-[(4,6-disubstitutedpyrimidine-2-yl) thio] acetohydrazides (4a-c), which on condensation with phenyl isothiocyanate in ethanol gave 2-{[(4,6-disubstituted pyrimidine-2-yl) thio] acetyl}-N-phenylhydrazinecarbothioamides (5a-c). The compounds (5a-c) on treatment with concentrated sulphuric acid yielded 5-{[(4,6-disubstituted pyrimidine-2-yl) thio] methyl}-N-phenyl-1, 3, 4-thiadiazol-2-amines (6a-c). All the compounds synthesized were characterized by their elemental analysis, IR and ^1^H NMR spectra. The physical and chemical data are presented in Table 1. In the IR spectrum of 2b, the presence of band at 2840 cm^−1^ (SH) and the absence of band due to >C=O confirmed the formation of pyrimidine-2-thiol moiety. The appearance of singlet at δ 9.72 due to SH also confirmed the formation of 2b. The IR spectrum of 5b exhibited band at 1681 cm^−1^ (>C=O), 1667 cm^−1^ (>C=N) and 3120-3218 cm^−1^ due to N-H. The ^1^H NMR spectrum of 5b exhibited the aromatic and heterocyclic protons as a multiplet integrating for 15 protons from δ 7.21-7.92 and a multiplet integrating for 3 protons from δ 8.20-10.12 due to NH.NH.CS.NH. In the IR spectrum of 6b, the disappearance of bands at 1681 cm^−1^ (>C=O) and 1453 cm^−1^ (>C=S) and the appearance of band at 746 cm^−1^ (C-S-C) confirmed the formation of thiadiazole ring. The ^1^H NMR spectrum of 6b exhibited the aromatic and heterocyclic protons as a multiplet integrating for 15 protons from δ 7.21-8.32 and a singlet at δ 9.3 integrating for one proton due to NH. In the mass spectra, the molecular ion peak at 483 (M^+^) also confirmed the formation of titled compound 6b.

Compound 2c, 2d, 2g and 6b showed moderate DPPH free radical scavenging activity while all other compounds were found to be less active. Compounds 2c, 2d and 6b showed moderate nitric oxide free radical scavenging activity and all other compounds were found to be less active. As shown in Table 2 compounds 2b, 2c and 6b exhibited significant activity against human breast cancer MCF-7 cell line, while compounds 2e and 2h showed moderate cytotoxicity.
